# Case in Which Secale Cornutum Appeared to Be Efficacious in Expelling Coagula from the Uterus; with Remarks

**Published:** 1826-06

**Authors:** William Mackenzie

**Affiliations:** Andersonian Professor of Anatomy and Surgery, and one of the Surgeons to the Glasgow Eye Infirmary.


**Art. V.—*Case in which Secale Cornutum appeared to be efficacious in expelling Coagula from the Uterus; with Remarks.*** By William Mackenzie, Andersonian Professor of Anatomy and Surgery, and one of the Surgeons to the Glasgow Eye Infirmary.

Between ten and eleven o'clock of the evening of the 1st current, I was called to visit Mrs. ——, who, about ten o'clock of the morning of the same day, had been delivered of twins, her first children. I was given to understand by the midwife in attendance, that the delivery had been rapid and easy, and that, soon after the second child was born, a large double placenta had been expelled entire. But the uterus continued extremely large, far up in the abdomen, and very hard. In the course of the forenoon, a large clot of blood had come away, but this had not altered the state of matters; and the midwife suspected the presence of a third child in the uterus.

I found the uterine tumor very hard to the hand laid upon the abdomen, of a globular and elevated form, very high in the abdomen, little (if any) less than the uterus is after the expulsion of a first twin, and while the second yet remains undelivered. As I had little doubt, however, that it was filled merely with coagulated blood, I hesitated for a little whether I should not at once introduce my hand into the cavity of the uterus, and remove the coagula, or try the effect of the secale cornutum.

I resolved on the latter; and, having weighed out fifty grains and bruised them, I ordered the whole to be infused for ten minutes in a cupful of hot water. One half of this infusion was administered, without any perceptible effect. After half an hour, the other half was given along with the bruised pieces of secale. This was immediately followed by expulsive efforts, and a very large quantity of coagulated blood was discharged. The uterus was reduced to its usual size and situation after parturition. The patient soon after fell asleep, and slept for four hours; and has since done well.

Two objections may be started to this case.

1. It may be objected, that I should have rather removed the coagula by the hand, introduced into the uterus. But I would reply, that this operation is attended, especially after the interval of twelve or thirteen hours from the time of delivery, with very considerable pain and irritation; and that this irritation is likely to concur with any other predisposing or exciting causes of inflammation which may be present.

2. It may be said, that the expulsion of the coagula did not depend on the influence of the secale, but might have been incidental, or an effect of the mere stimulus of hope. I admit that I assured the patient that I had much confidence in the medicine which I was about to give to her. But that the event was incidental, does not appear probable, when we consider that twelve hours had passed without the uterus contracting to its ordinary size, and that for seven or eight hours no expulsive efforts whatever had taken place.

In a course of lectures on the Materia Medica, which I lately attempted, I placed the secale cornutum among the *nervous excitants*. The phenomena of convulsive ergotism appeared to justify my thus classifying this substance among the medicines which affect the muscular system, through the medium of nervous excitation. This view of the secale cornutum, if correct, may also serve to explain, in some measure, its effects in parturition, which is an action, not of the uterus alone, but also (nay chiefly) of the diaphragm and abdominal muscles.

An American graduate has asserted that the secale cornutum, in whatever manner exhibited, has little or no operation on the system of the male. The pulse, he says, is neither elevated nor depressed by it; and, excepting some nausea or vomiting from large doses, no other effect is manifested. To the uterus, he supposes, its whole force is exclusively directed. This notion is contradicted by the phenomena of convulsive ergotism, such as they have been observed and described by many authors. This disease, as described by Srinc, began with a disagreeable sensation in the feet, a kind of titillation or formication, speedily followed by strong contraction of the fingers, pain in the head, vertigo, delirium, spasms in the limbs, and opisthotonos. Of this disease, as observed by that author, many died, especially children. It evidently existed in the muscular system generally, and there can be no doubt that it affected males as well as females.*

So similar are the symptoms of convulsive ergotism to the phenomena displayed after a dose of nux vomica, and some other of the nervous excitants, that it is extremely probable there exists in the secale cornutum some alkaloid principle, analogous to strychnia.

*Spreull's-court; 21st April*, 1826.

---

* See Tissot's Letter to Sir George Baker, in the Philosophical Transactions, vol. lv. page 113, for an account of the different kinds of ergotism, and the instances of their occurrence.

